# Correction: Pandemic-associated mobility restrictions could cause increases in dengue virus transmission

**DOI:** 10.1371/journal.pntd.0011032

**Published:** 2023-01-04

**Authors:** Sean M. Cavany, Guido España, Gonzalo M. Vazquez-Prokopec, Thomas W. Scott, T. Alex Perkins

There is an error in Fig 3. The values on the color bar for picture C. Population density /km^2^ are in correct. Instead of ranging from 0 to 1000, they should range from 5000 to 25000. Please see the correct Fig 3 here.

**Fig 3:**
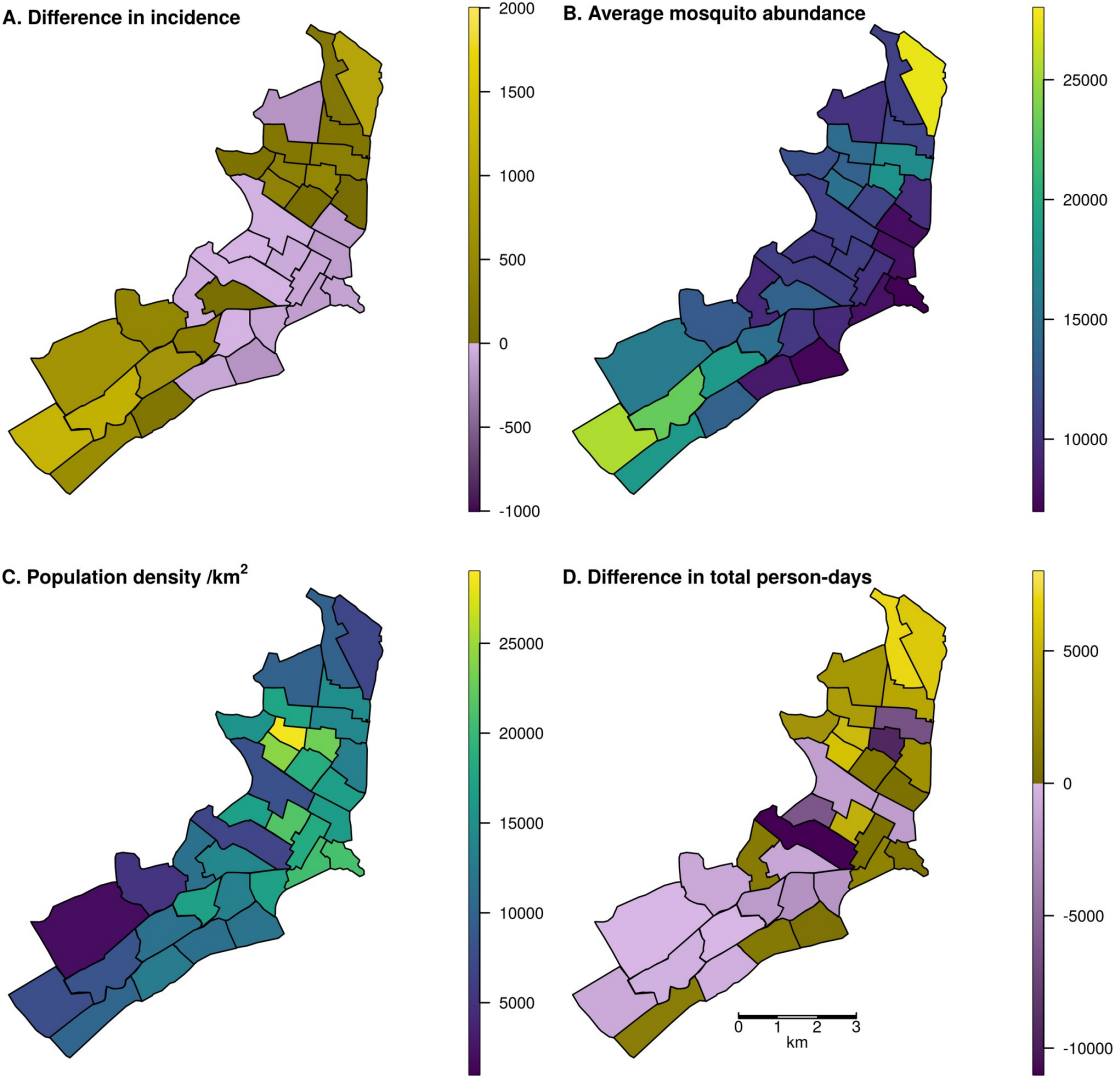
Map of Iquitos, with the 35 Ministry of Health (MoH) zones delineated. In panels A and D, yellow indicates increases and blue indicates decreases. In panels B and C, colors are a continuous scale showing the given metric. A: Spatial distribution of changes in total incident DENV infections, assigned to the home zone of the infected individual, across a two-year period including the serotype invasion and following seasons. Lockdown was initiated on March 17 in the serotype invasion season. B: Total mosquito abundance across different MoH zones, averaged across the two-year period. C: Human population density of the MoH zones. D: Difference in the total person-days spent in each zone between lockdown and baseline scenarios assuming 70% of people complied with lockdown measures. Shape files for the underlying maps can be found at github.com/scavany/dengue_shelter_in_place.
