# Supplementary material for: Pandemic-associated mobility restrictions could cause increases in dengue virus transmission
Source: PLoS Negl Trop Dis. 2021 Aug 9;15(8):e0009603. doi: 10.1371/journal.pntd.0009603 (PMC8375978; doi:10.1371/journal.pntd.0009603)
Supplement: S1 Text — (PDF) [file pntd.0009603.s001.pdf]

S1 Text

Our agent-based model (ABM) includes both people and mosquitoes as agents. These agents interact with each other in an environment represented by a set of locations, including houses, schools, parks, cemeteries, and churches. This environment also incorporates temporally varying climatic conditions, which affect mosquito biting frequency, survival, and extrinsic incubation period for dengue virus (DENV). The environment is based on the city of Iquitos in Peru, and we represent all 92,891 buildings in the city. We use exact spatial coordinates and location type for the 38,835 locations for which these data were available. For locations without these data, we randomly distributed the locations and assigned a location type so that they were evenly spaced and representative of the location types we had data on.

We modeled approximately 450,000 humans in our model, chosen to reflect the demography of Iquitos and its surroundings [1]. We based the overall age and sex distribution on U.N. estimates of these for Peru, and based the demographic profile of each household on survey data from a prior study [2]. This synthetic population also realistically captured how people are distributed across houses and how demographics changed over time. We achieved this by simulating human births and deaths that match those estimated by the U.N. for Iquitos and simultaneously preserved realistic household compositions by placing newborn children in houses with appropriately aged mothers as determined by U.N. estimates of age-specific fertility of Peru [1]. All human agents in the model went through daily human movement patterns using a model previously described by Perkins et al. [3], except when this was modified by lockdown policies which meant they stayed home (see Methods). To describe these movement patterns, each agent has five daily movement trajectories. At the start of each day one of these five trajectories is chosen, with equal probability, for each agent. The human movement patterns were fitted to data from retrospective, semi-structured interviews with inhabitants of Iquitos [4,5].

Immature mosquitoes were modeled deterministically and independently at each location. They transitioned through three immature stages: eggs, larvae, and pupae, with the number of pupae in a

house determining the rate of emergence of adult mosquitoes in that house. The rate of transition between each of these stages was temperature dependent. All stages also underwent temperature-dependent mortality. Larval stages underwent an additional density-dependent mortality [6,7]. Both the larval and pupal stages also underwent an additional rate of mortality that was calibrated so that adult abundance matched a statistical estimate of the spatio-temporal adult abundance in Iquitos [8]. Adult mosquitoes are modeled as agents, and take blood-meals upon co-located human agents. When a mosquito takes a blood-meal, the time of its next blood-meal is determined according to an exponential distribution with a temperature-dependent gonotrophic rate parameter, based on temporal trends in temperature and empirical relationships between these rates and temperature [7,9]. The daily gonotrophic rate is

$$d_i(T)=\frac{\rho_i \frac{T}{298}\exp\left(\frac{\Delta_{A,i}}{R}\left(\frac{1}{298}-\frac{1}{T_{W,mean}}\right)\right)}{1+\exp\left(\frac{\Delta_{H,i}}{R}\left(\frac{1}{T_{1/2}}-\frac{1}{T_{W,mean}}\right)\right)} \quad ,$$

where T is the daily mean temperature in Kelvin and all other parameters are given in S1 Table. When the mosquito's next blood-meal is due, it will take it unless there is no human present. This means that the number of blood-meals taken by a mosquito is not determined by the local density of humans, except in the unusual instance in which no human is present at a location. The mosquito determines which human it will bite as a function of the body sizes of humans present at that time – the mosquito chooses who to bite with probability proportional to the surface-area of individuals in the building at that time [2]. Each day, each mosquito moves to another location with probability 0.3, and will only move to a location within 100 m of its starting location, consistent with another agent-based model of *Ae. aegypti* population dynamics [6].

S1 Table: definition of parameters governing gonotrophic rate

Parameter	***Definition***	***Gonotrophic cycle***
ρ_i	Development rate per hour at 25ºC assuming no temperature inactivation of	0.00898

	the critical enzyme (hr^{-1})	
$\Delta_{A,i}$	Enthalpy of activation of the reaction catalyzed by the enzyme (cal/mol)	15,725.23
$\Delta_{H,i}$	Enthalpy change associated with high temperature inactivation of the enzyme (cal/mol)	1,756,481.07
$T_{1/2}$	Temperature at which 50% of the enzyme is inactivated from high temperature	447.17

When a mosquito takes a blood-meal on a human and one of them is infected, transmission can occur. The probability that transmission occurs from humans to mosquitoes is determined by the time since the human was infected, with this probability based on the viremia levels of the infecting human at the time of the bite [10]. For transmission from mosquitoes to humans, infectious mosquitoes transmitted DENV to susceptible humans with a fixed probability of 1.0, providing the extrinsic incubation period has been completed [11,12]. Once infected humans develop symptoms following a latent period derived from timing of peak viraemia [11]. Following recovery, humans become permanently immune to that serotype and also have temporary heterologous immunity to all other serotypes. This temporary immunity lasts for an exponentially distributed period with mean of 686 days, estimated in a previous modeling study of time-varying serotype-specific dengue incidence [13]. The initial level of immunity in the model was calibrated to an analysis of longitudinal serological data [14].

Supplementary References

1. Perkins TA, Reiner RC, España G, Ten Bosch QA, Verma A, Liebman KA, et al. An agent-based model of dengue virus transmission shows how uncertainty about breakthrough infections influences vaccination impact projections. PLoS Comput Biol. 2019 Mar 20;15(3):e1006710.
2. Liebman KA, Stoddard ST, Reiner RC, Perkins TA, Astete H, Sihuincha M, et al. Determinants of Heterogeneous Blood Feeding Patterns by Aedes aegypti in Iquitos, Peru. Barrera R, editor. PLoS Neglected Tropical Diseases. 2014 Feb 13;8(2):e2702.
3. Perkins TA, Garcia AJ, Paz-Soldán VA, Stoddard ST, Reiner RC, Vazquez-Prokopec G, et al. Theory and data for simulating fine-scale human movement in an urban environment. J R Soc Interface [Internet]. 2014 Oct 6 [cited 2018 Oct 5];11(99). Available from: https://www.ncbi.nlm.nih.gov/pmc/articles/PMC4233749/
4. Paz-Soldan VA, Stoddard ST, Vazquez-Prokopec G, Morrison AC, Elder JP, Kitron U, et al. Assessing and maximizing the acceptability of global positioning system device use for studying the role of human movement in dengue virus transmission in Iquitos, Peru. Am J Trop Med Hyg.

2010 Apr;82(4):723–30.

5. Paz-Soldan VA, Reiner RC, Morrison AC, Stoddard ST, Kitron U, Scott TW, et al. Strengths and weaknesses of Global Positioning System (GPS) data-loggers and semi-structured interviews for capturing fine-scale human mobility: findings from Iquitos, Peru. PLoS Negl Trop Dis. 2014 Jun;8(6):e2888.
6. Magori K, Legros M, Puente ME, Focks DA, Scott TW, Lloyd AL, et al. Skeeter Buster: a stochastic, spatially explicit modeling tool for studying Aedes aegypti population replacement and population suppression strategies. PLoS Negl Trop Dis. 2009 Sep 1;3(9):e508.
7. Otero M, Solari HG, Schweigmann N. A stochastic population dynamics model for Aedes aegypti: formulation and application to a city with temperate climate. Bull Math Biol. 2006 Nov;68(8):1945–74.
8. Reiner RC, Stoddard ST, Vazquez-Prokopec GM, Astete H, Perkins TA, Sihuincha M, et al. Estimating the impact of city-wide Aedes aegypti population control: An observational study in Iquitos, Peru. PLoS Negl Trop Dis. 2019 May;13(5):e0007255.
9. Focks DA, Haile DG, Daniels E, Mount GA. Dynamic Life Table Model for Aedes aegypti (Diptera: Culicidae): Analysis of the Literature and Model Development. J Med Entomol. 1993 Nov 1;30(6):1003–17.
10. Ten Bosch QA, Clapham HE, Lambrechts L, Duong V, Buchy P, Althouse BM, et al. Contributions from the silent majority dominate dengue virus transmission. PLoS Pathog. 2018 May;14(5):e1006965.
11. Chan M, Johansson MA. The incubation periods of Dengue viruses. PLoS ONE. 2012;7(11):e50972.
12. Ellis AM, Garcia AJ, Focks DA, Morrison AC, Scott TW. Parameterization and Sensitivity Analysis of a Complex Simulation Model for Mosquito Population Dynamics, Dengue Transmission, and Their Control. Am J Trop Med Hyg. 2011 Aug 1;85(2):257–64.
13. Reich Nicholas G., Shrestha Sourya, King Aaron A., Rohani Pejman, Lessler Justin, Kalayanarooj Siripen, et al. Interactions between serotypes of dengue highlight epidemiological impact of cross-immunity. Journal of The Royal Society Interface. 2013 Sep 6;10(86):20130414.
14. Reiner RC, Stoddard ST, Forshey BM, King AA, Ellis AM, Lloyd AL, et al. Time-varying, serotype-specific force of infection of dengue virus. Proc Natl Acad Sci USA. 2014 Jul 1;111(26):E2694-2702.
