# Supplementary figures and images for: Pandemic-associated mobility restrictions could cause increases in dengue virus transmission
Source: PLoS Negl Trop Dis. 2021 Aug 9;15(8):e0009603. doi: 10.1371/journal.pntd.0009603 (PMC8375978; doi:10.1371/journal.pntd.0009603)

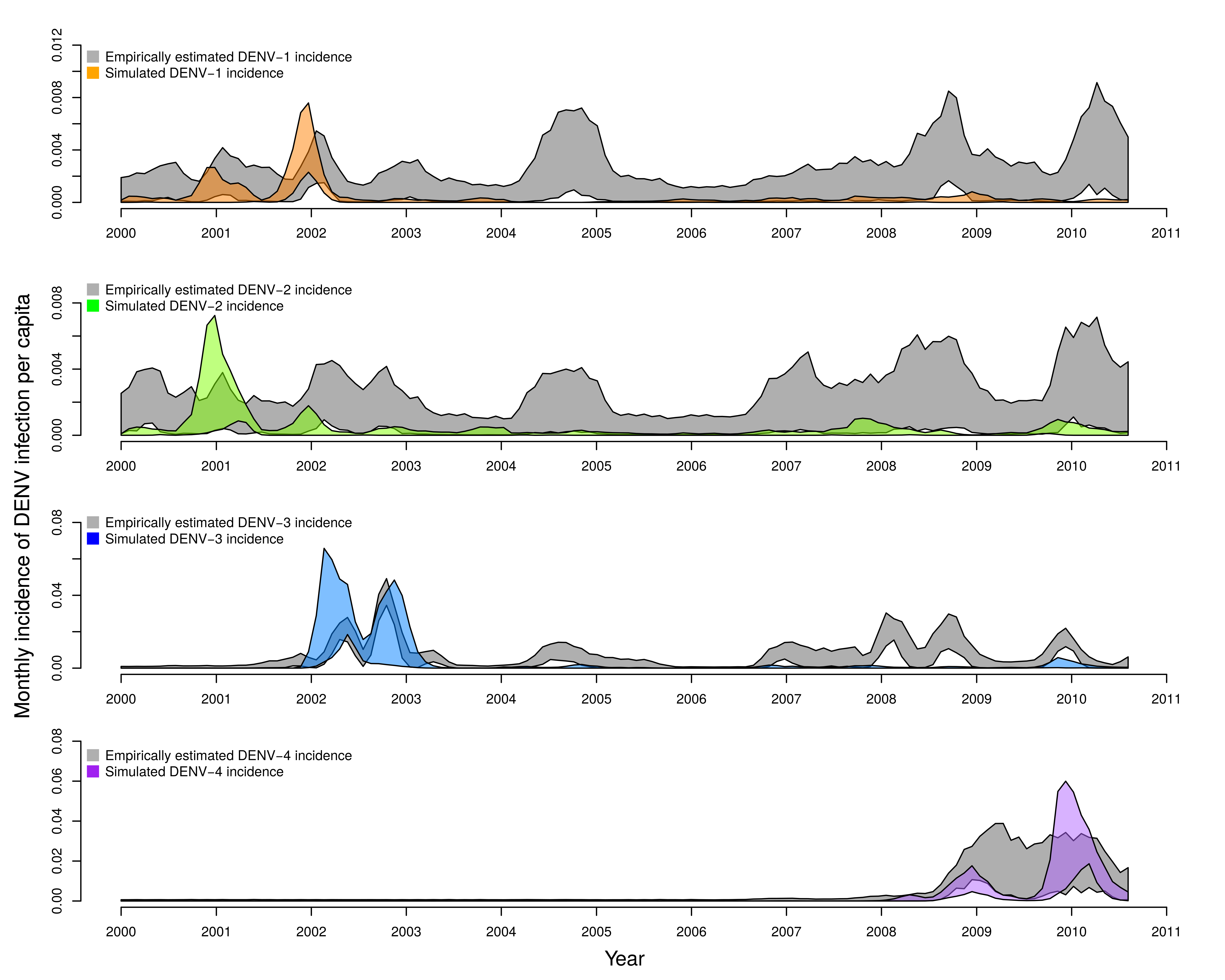

Supplement: S1 Fig — Taken from [27] (TIF) [file pntd.0009603.s002.tif]

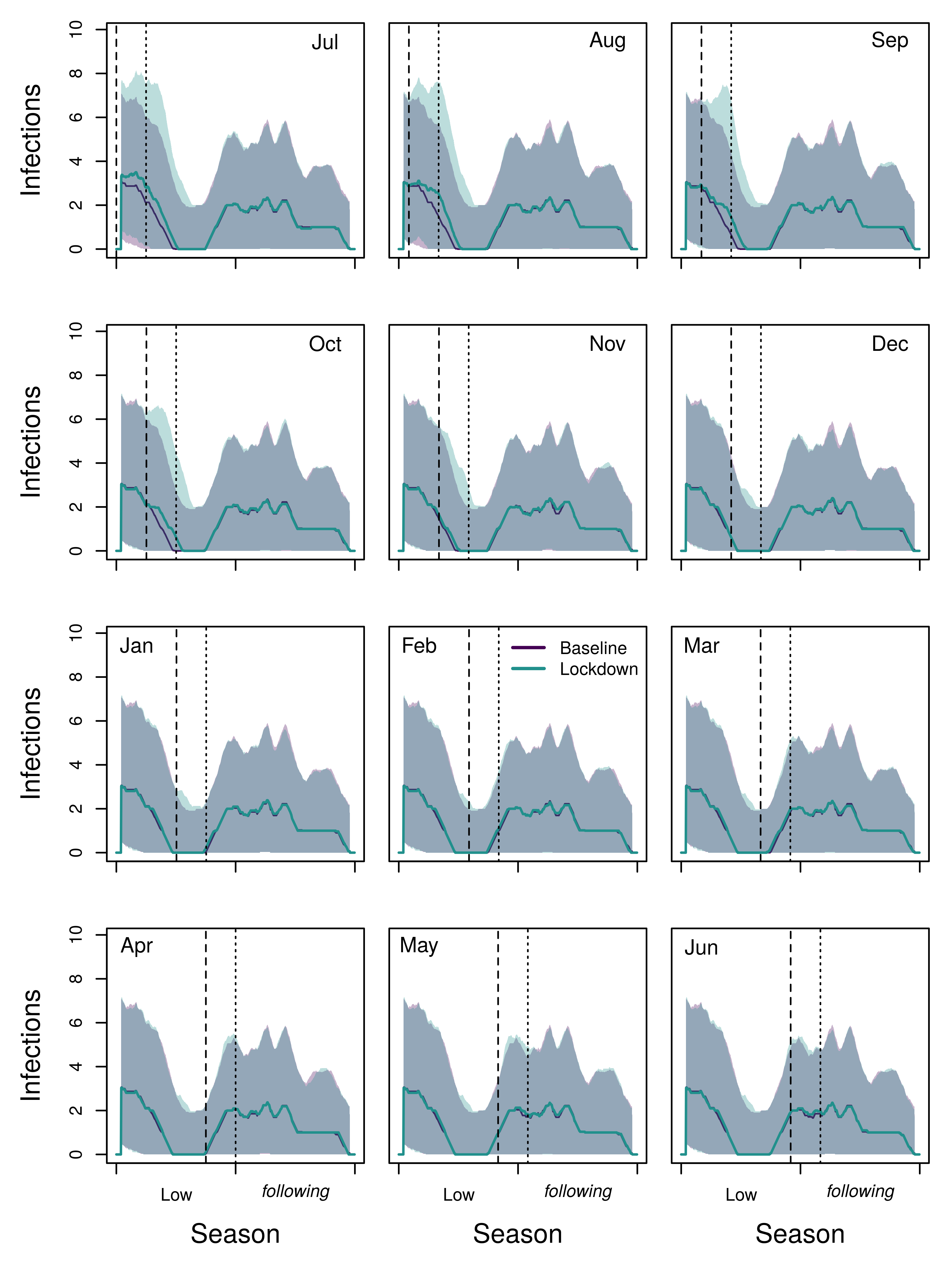

Supplement: S2 Fig — Shaded regions are the interquartile range. Shading in gray is where these regions overlap. (TIF) [file pntd.0009603.s003.tif]

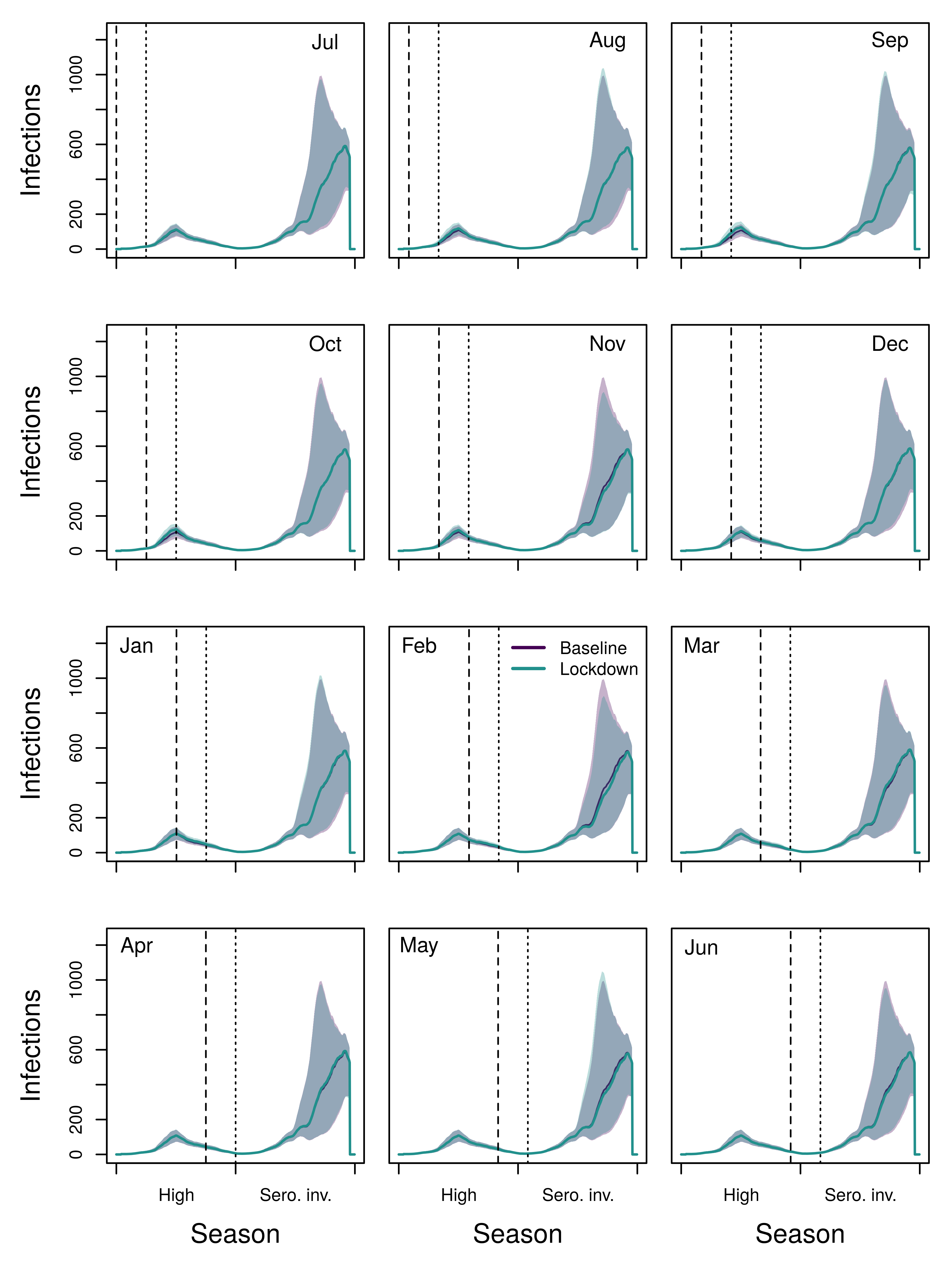

Supplement: S3 Fig — Shaded regions are the interquartile range. Shading in gray is where these regions overlap. (TIF) [file pntd.0009603.s004.tif]

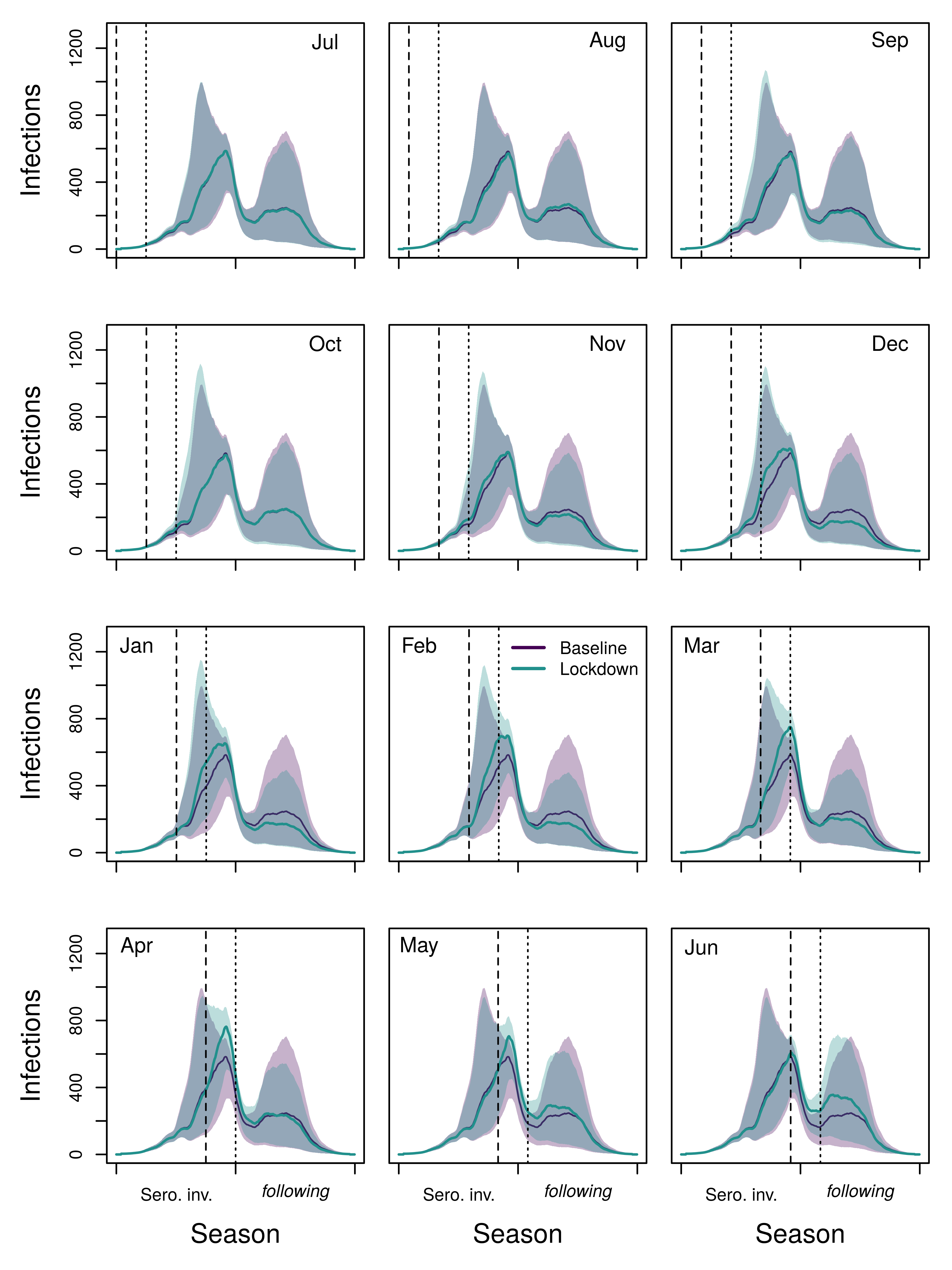

Supplement: S4 Fig — Shaded regions are the interquartile range. Shading in gray is where these regions overlap. (TIF) [file pntd.0009603.s005.tif]

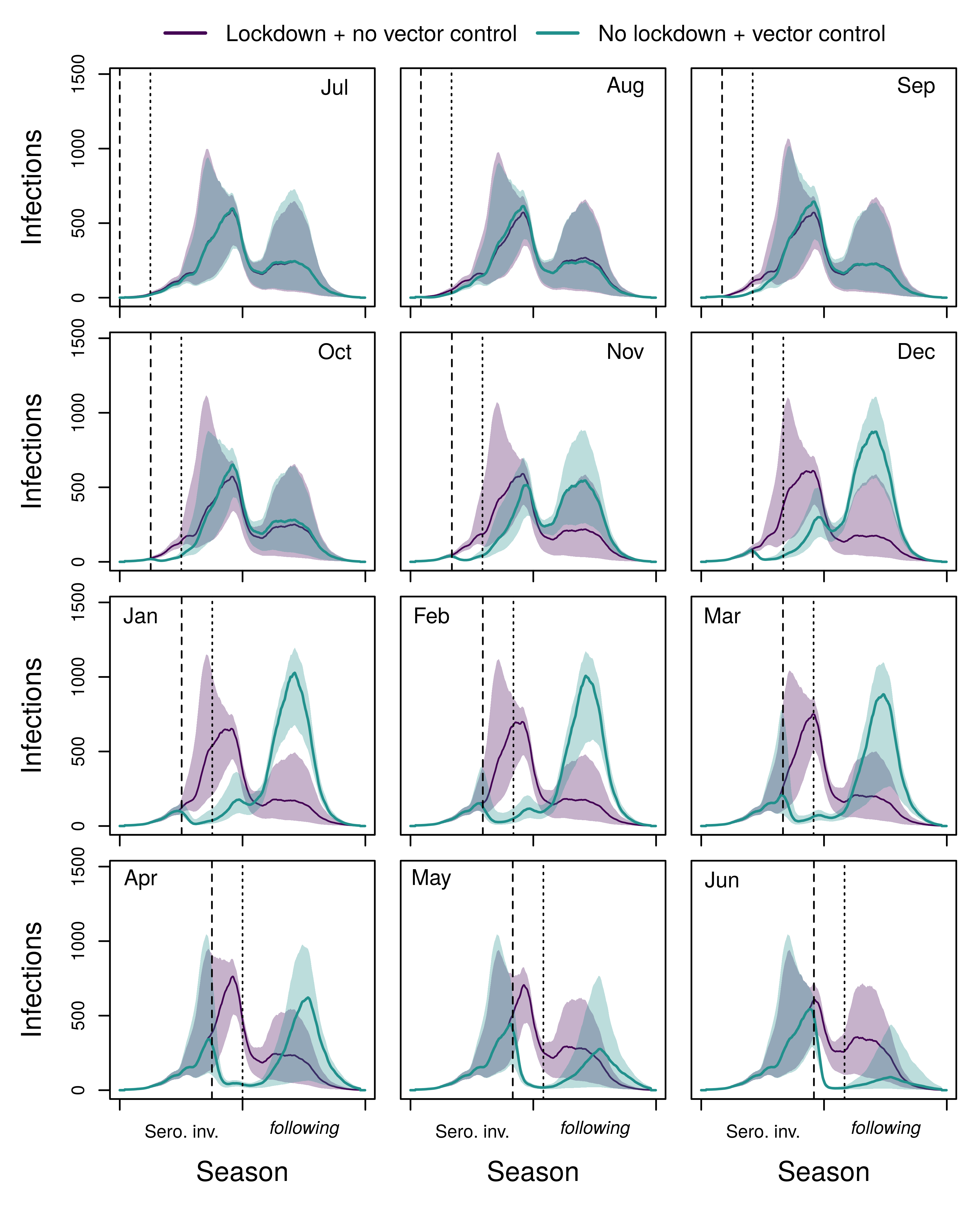

Supplement: S5 Fig — Lockdown and the city-wide vector control campaign began at the dashed line. Lockdown lasted three months (ending at the dotted line). The vector control campaign lasted three weeks. Shaded regions are the interquartile range. Shading in gray is where these regions overlap. (TIF) [file pntd.0009603.s006.tif]

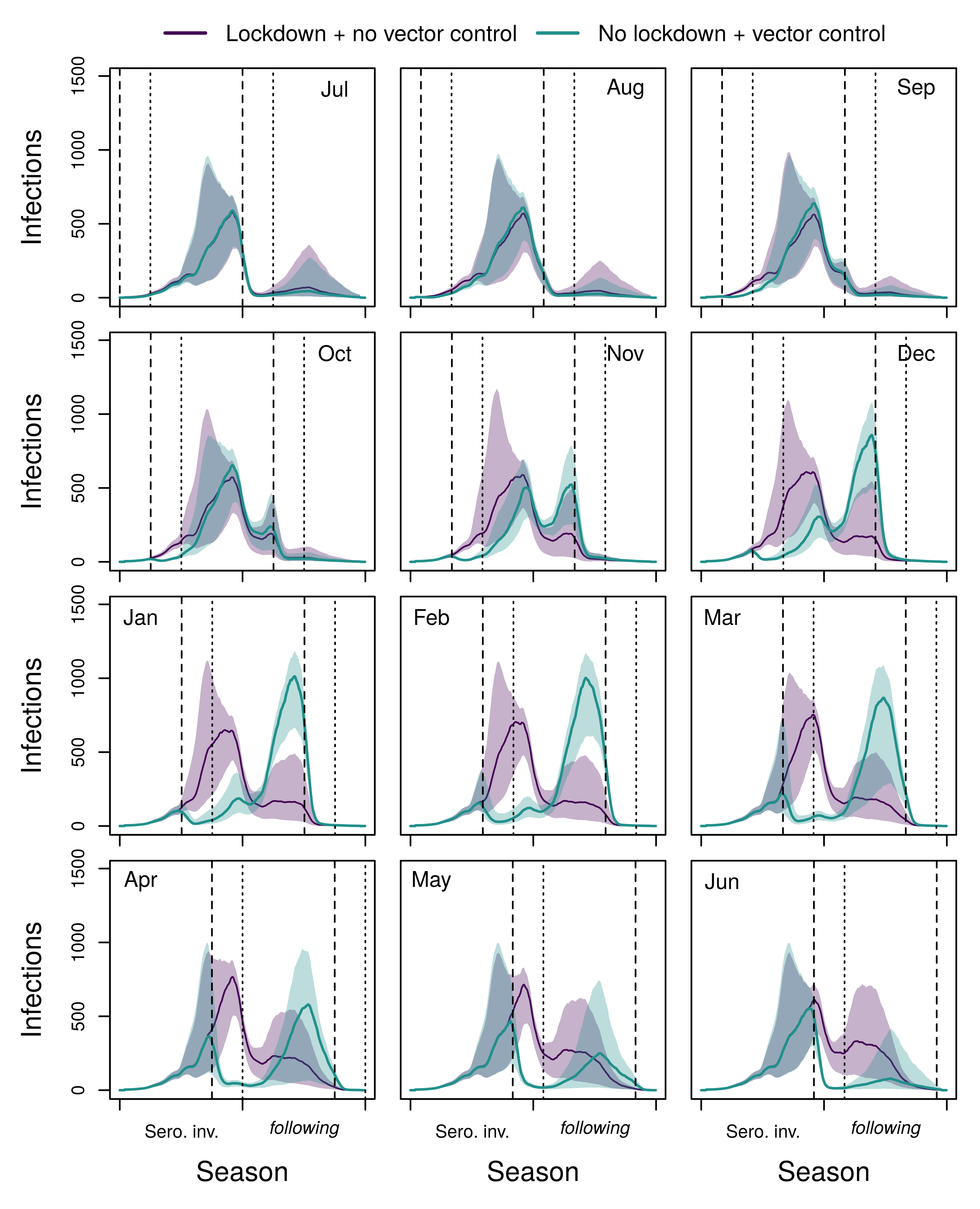

Supplement: S6 Fig — Both lockdown and the city-wide vector control campaign began at the dashed line in the first season. In the following season, vector control occurred in both simulations and lockdown did not occur in either simulation. Lockdown lasted three months (ending at the dotted line). The vector control campaign lasted three weeks. Shaded regions are the interquartile range. Shading in gray is where these regions overlap. (TIF) [file pntd.0009603.s007.tif]

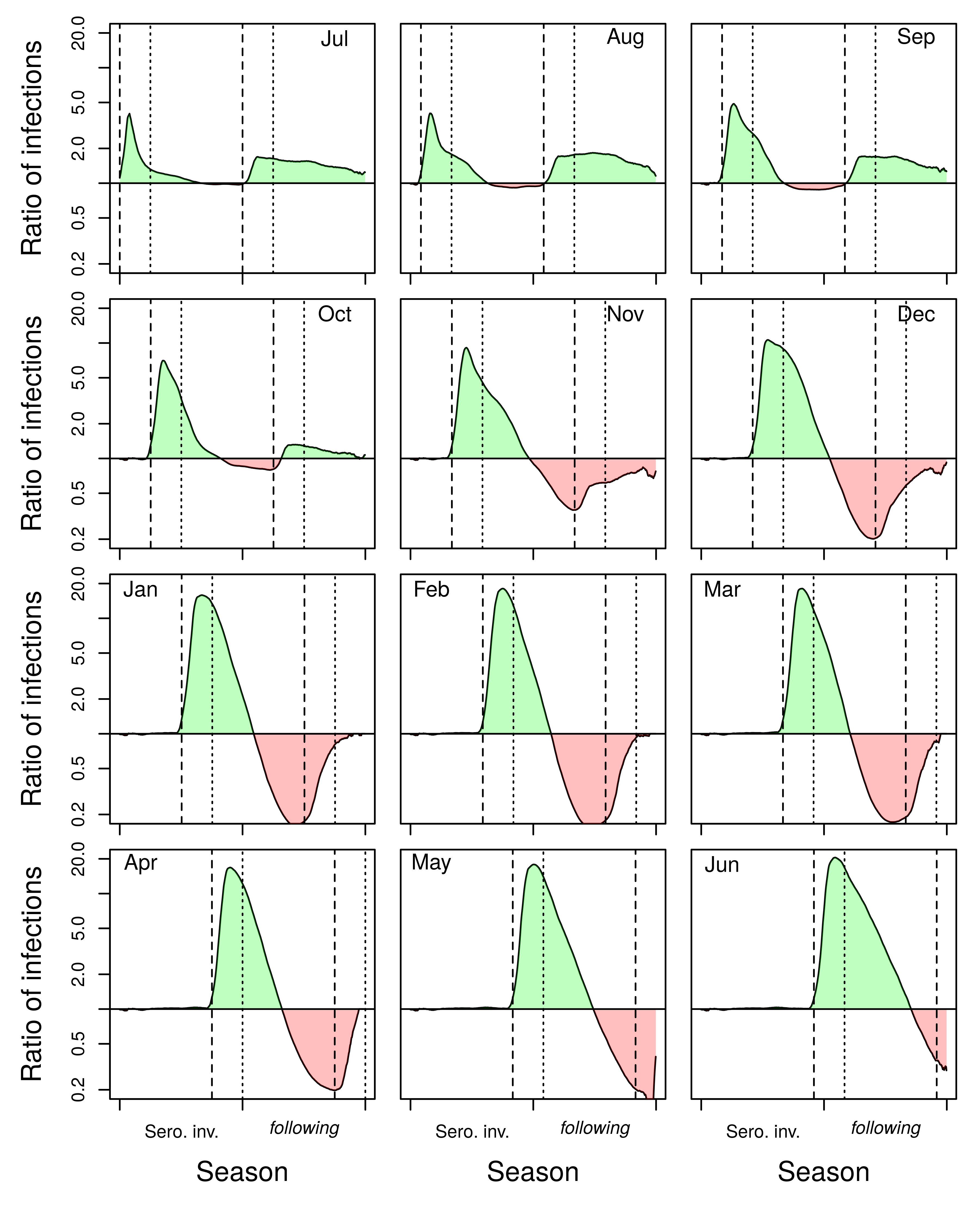

Supplement: S7 Fig — Lockdown began at the vertical dashed line, and ended at the dotted line. In the following season, vector control occurred in both simulations and lockdown did not occur in either simulation. (TIF) [file pntd.0009603.s008.tif]

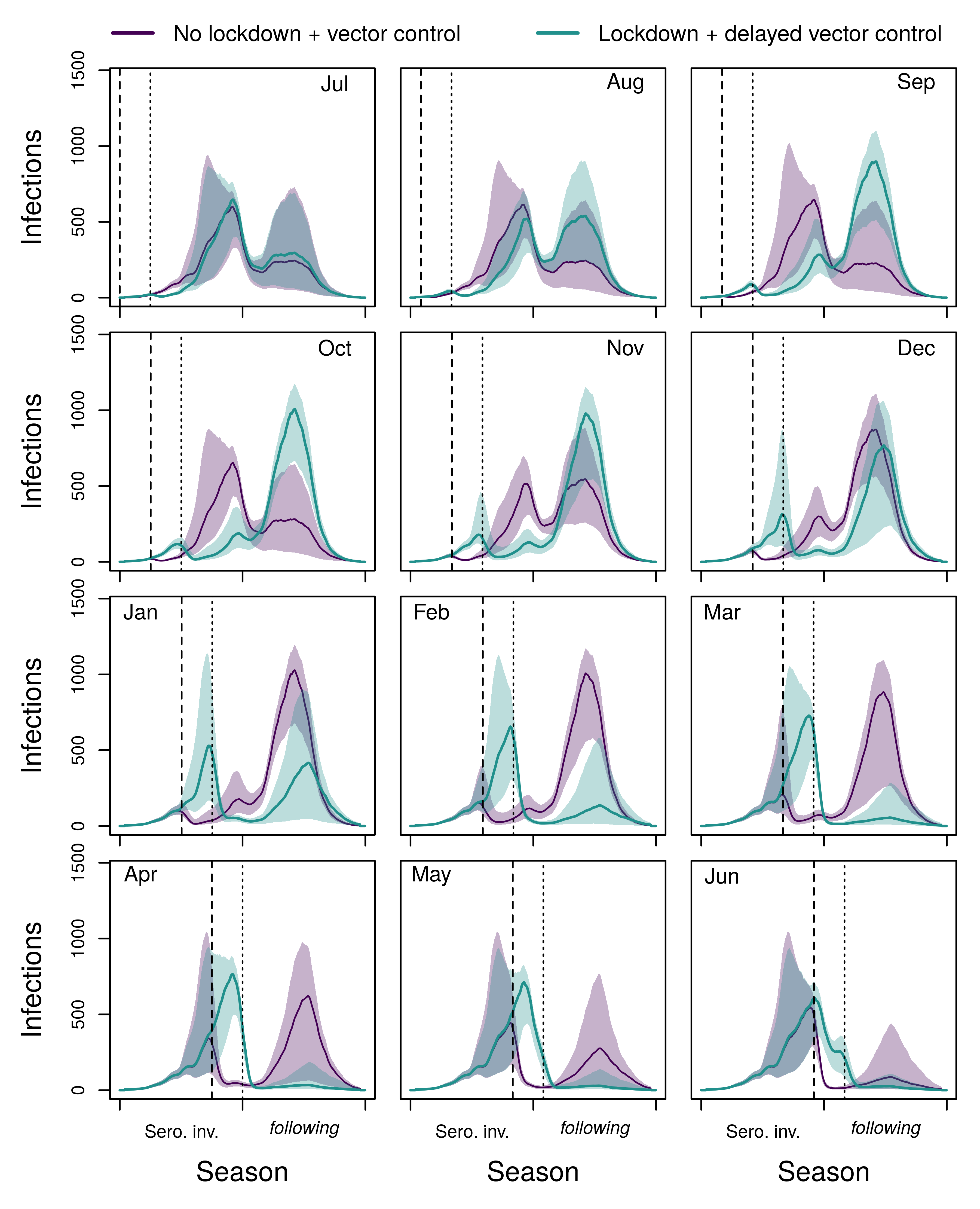

Supplement: S8 Fig — Lockdown lasted three months, starting at the dashed line and ending at the dotted line. The vector control campaign lasted three weeks, beginning at the dashed line (purple) or the dotted line (green). Shaded regions are the interquartile range. Shading in gray is where these regions overlap. (TIF) [file pntd.0009603.s009.tif]

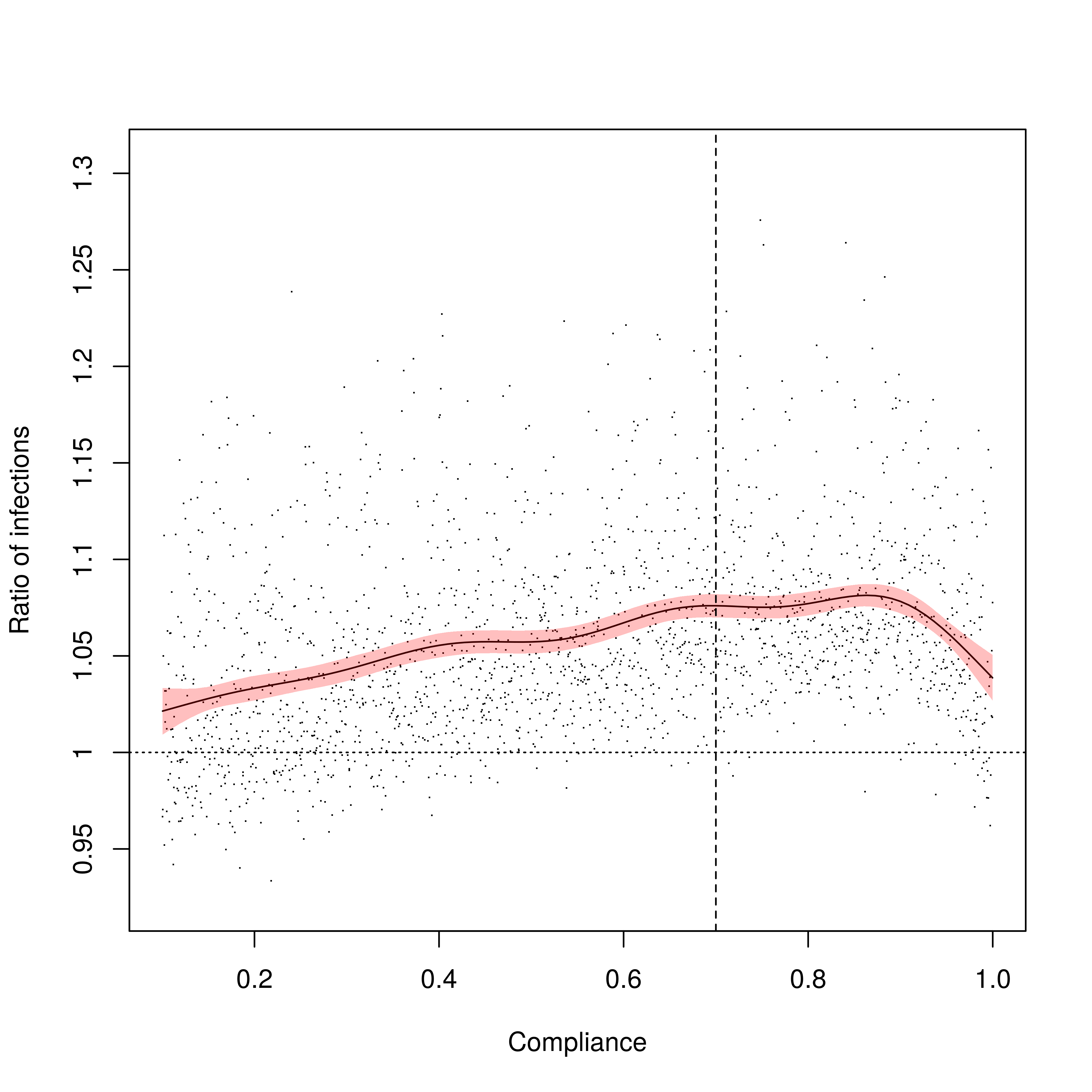

Supplement: S9 Fig — The vertical dashed line shows baseline compliance (70%). The horizontal dashed line shows when there is no effect of lockdown. (TIF) [file pntd.0009603.s010.tif]

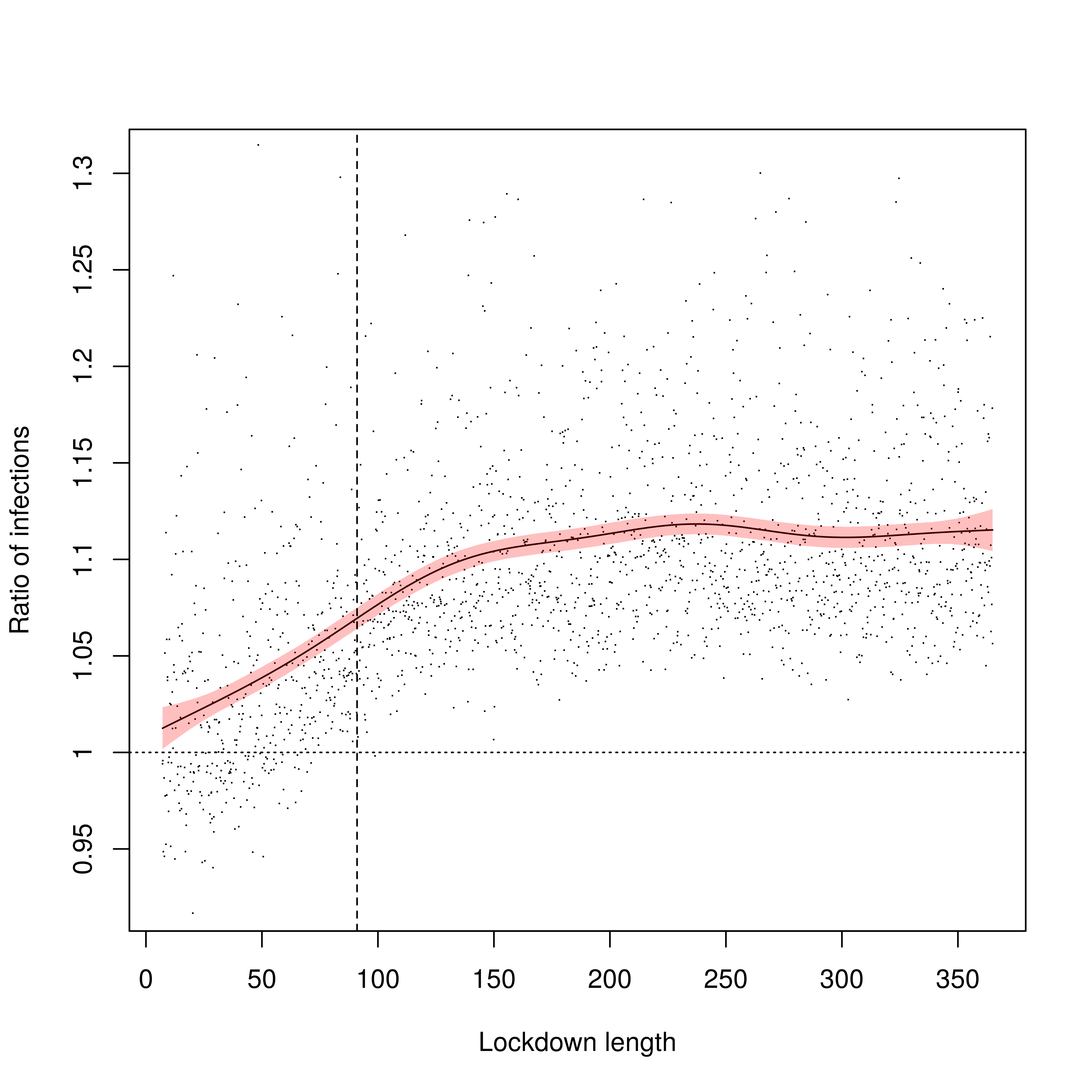

Supplement: S10 Fig — The vertical dashed line shows the baseline length (three months). The horizontal dashed line shows when there is no effect of lockdown. (TIF) [file pntd.0009603.s011.tif]
