# Supplementary material for: Pandemic-associated mobility restrictions could cause increases in dengue virus transmission
Source: PLoS Negl Trop Dis. 2021 Aug 9;15(8):e0009603. doi: 10.1371/journal.pntd.0009603 (PMC8375978; doi:10.1371/journal.pntd.0009603)
Supplement: Attachment — Submitted filename: shelter_in_place_dengue_PLOS_NTDs_response_to_reviewers.docx [file pntd.0009603.s012.docx]

**Editors' comments:**

I have read the comments and would ask that you pay particular attention to justifying the timing of the lockdown, as well as provide justification/ referencing for some of the parameter values. Additionally, the language in the discussion should be softened the reflect the assumptions and repercussions thereof of the model.

We thank the editor and reviewers for their comments. Specific concerns have been addressed below. Line numbers refer to the tracked changes version.

**Reviewer's Responses to Questions**

**Methods**

**Reviewer #1:** Generally the objectives and methods are clear. A few questions below:

1. lines 103-104 How could this calibration effect the results of this study? for example, have movement within and into Iquitos and/or populations, buildings, etc. change significantly since the early 2000/s, especially during the pandemic? How would this affect the outcomes?

Response: We added a comment to the discussion to address the potential limitation of not considering changing importation patterns (lines 390-395).

*“Third, we did not assess the impact of changing importation patterns into Iquitos. As our model predicts the biggest impact of lockdown is late in the season, a time when the epidemic is predominantly driven by local transmission, we would not expect changes in imported infections to qualitatively affect this finding. However, changing importations may have a significant impact if they prevent an imported infection seeding a new outbreak, particularly if this was with a new serotype.”*

2. lines 108-109 Does this mean you are assuming that 30% of the people have essential jobs? Or that 70% of the people without essential jobs are staying home? Also, this doesn't necessarily need to be included in this study, but it are the essential workers more likely to come from certain parts of the city or higher incidence neighborhoods?

Response: What we mean is closer to the former – 30% of people undertake their normal movement pattern, either because they do not comply, or because they have an essential job. This has been clarified in the text (lines 115-117).

*“That is, 30% of individuals do not comply with lockdown, either because they choose not to or because they have an essential job.”*

3. lines 121-123 It's not always clear when vector control is applied versus not. For example, did the original calibration include vector control?

Response: Vector control did not take place during the calibration, nor the baseline simulations. This has been clarified in the text (lines 130-131).`

*“In baseline simulations and for the calibration, vector control did not take place.”*

4. Figure 1, is there a comparison with the reported case counts and the simulated data?

Response: Details of the model fit are provided in the two prior papers, specifically Fig 3 of Perkins et al and S1 Fig of Cavany et al. A reference to this has been added to the text in lines 109-110.

*“See Fig 3 in Perkins et al. and S1 Fig in Cavany et al. for a visual representation of the calibration.”*

5. Line 144 What does "without simulating the full model" mean?

Response: This means we are analyzing the movement trajectories for each individual, comparing their baseline trajectory with one edited so that individuals spend all of their time at home. Thus, these movement trajectories are inputs to the transmission model and were not run during simulations involving transmission. We amended the text to clarify this (line 157-160):

“The movement trajectories are calculated prior to simulating the agent-based models, and are then used as inputs to the model. By comparing the movement trajectories directly, i.e. without simulating the model, we analyzed the effect of changing these trajectories so that people spend all of their time at home.”

6. Line 148 why is the unique number of visitors greater under lockdown? wouldn't there be the same household members at home without extra people visiting?

Response: We had been calculating this metric by averaging across the five movement trajectories. As some individuals have a movement trajectory where they are away from home for an entire 24 hour period, this meant that in a small minority of cases (1%) the average number of unique visitors on a given day could go up in lockdown as those individuals would then be home for that 24-hour period instead.

However, following your comment, we decided that it would be more fair to estimate the metric as the total number of unique visitors across all the movement trajectories. However, as you point out, the metric calculated this way would be trivial as it could only decrease under lockdown. So, we decided instead to remove this metric from the analysis completely and focus solely on the average number of people, not the number of unique people.

7. Line 152 I assume that "compliance" is referring to lockdown and not the vector control compliance referenced earlier?

Response: Yes, you are correct, this refers to lockdown compliance. This has been clarified in the text (line 167)

**Reviewer #2**: The hypothesis of the study is clear and the study design is appropiate.

Methodology is appropiate but it is necessary to search for other papers to fully understand it.

Minor comment: In the line 98-99 is stated that “Each mosquito determines when to bite based on a temperature dependent rate parameter”, I would like to know how other variables that are known to influence feeding like gonotrophic cycle or body size are involved in this part. It is also not very clear in the supplementary material.

Response: Apologies that the methodology is described across several manuscripts. Because the model is quite complex and has been developed over several years this is sadly unavoidable, although we aim to have included all relevant details in this paper and its supplementary materials.

We added more details to the supplementary material describing when and who the mosquito will bite. In particular, we added the equation governing the gonotrophic cycle rate to line 44, and added the following line to line 51 describing who the mosquito chooses to bite:

*“the mosquito chooses who to bite with probability proportional to the surface-area of individuals in the building at that time”*

We also added a note to line 104 in the main text directing the reader to the supplementary material for additional details on this matter.

**Reviewer #3**: See general comments. Major issues with study design

**Results**

**Reviewer #1**: The results match and presentation is generally clear. A few questions below.

1. Lines 163-164 Interesting that one month difference in timing makes such a big difference in results. Is this June lockdown referred to here a month before the July lockdown or 11 months later in the simulation? I guess more specifically, is it right before the introduction of a new serotype or 11 months after? From looking at the first figure, June is normally low incidence but happened to be particularly high because of the timing of the serotype invasion that season which allowed transmission even during a normally lower mosquito density because of the high susceptibility of the human hosts. So it's not so much the particular month as it is whether or not the lockdown is happening when incidence is over some kind of high threshold. Is that a correct interpretation?

Response: You are correct, the important factor seems to be whether lockdown occurs when prevalence and the force of infection are at their highest. We added text to the discussion in order to explain this observation (lines 305-309).

*“Though the increase was relatively modest, the effect was most pronounced when lockdown occurred during periods of time when the prevalence of infection, and hence the force of infection, was highest. In the scenarios considered in this study, that scenario occurred at the end of a novel serotype invasion season, following several months of heightened transmission.”*

2. Figure 2 says you are including two years worth of incidence in the plot but this wasn't clear from the Methods section or in other descriptions--whether or not you are considering the one season or multiple in the results.

Response: The caption is correct – incidence is over two years. We clarified this in both the methods (lines 148-150) and results (lines 171, 174)

*“Where cumulative incidence is reported in the results, it is over two seasons: the season of the lockdown and the following season. When location is shown, we assign infections to the home of the infected individual.”*

3. Line 173 "spatial distribution" Are you referring to the location where infectious bite occurred or the home of the person infected? (This applies to the spatial maps of infection as well)

Response: When location is reported, it is the home of the person. This applies to the map, too. We clarified this in the text (150, 186, 211)

*“When location is shown, we assign infections to the home of the infected individual.”*

4. Lines 186-189 Not sure that it makes sense to have higher incidence with fewer people-hours. More explanation would be helpful. Were incidence increases were in people residing there? Or people infected while visiting there?

Response: This is due to plotting the home of the person infected. So, there was a decrease in person-hours as fewer people were visiting this location. However, due to those people who live in this district spending more time there and the high mosquito abundance in homes there, transmission in those homes increases. We added the following explanation to the text (lines 206-208)

*“This is due to the fact that while fewer people were visiting this region, resulting in an overall decrease in person-hours, the people who live in this region were spending more time in homes with relatively high mosquito abundance.“*

5. Lines 208-214 These numbers are confusing. It says 78,562 buildings have more people inside them but then says 9,761 residential locations had higher numbers of people during lockdown

Response: This is a typo, the second part should say lower. It is now corrected.

6. Lines 217-225 It's not clear when vector control is occurring or not in these descriptions.

Response: This is not with vector control – we clarified this in the text (line 239).

7. Figure 5. How does the movement rate determined in the original calibration compare to the numbers shown in the plot? is there reason to think that mosquito movement increased or is this more of a "what-if" scenario?

Response: In the original calibration and most simulations, a value of 0.3 was used (the vertical line in the plot). These simulations are intended to explore the role of mosquito movement in a lockdown situation, rather than to suggest that mosquito movement may have changed under lockdown.

8. Lines 293-295 Why does more homogeneity result in fewer unique bites? This seems counter-intuitive

Response: Actually, more homogeneity results in more unique bites, as you expect. We added an extra line to explain this further (324-327).

*“lockdown results in a more homogeneous distribution of person-hours across locations, which leads to an increase in the number of unique individuals that each mosquito bites because there are fewer mosquitoes with no-one to bite and many mosquitoes have more options of who to bite.“*

**Reviewer #2**: Results do match analysis plan.

Some comments:

Lines 179-181: “The correlation between cumulative incidence and average mosquito abundance was r = 0.925 when no lockdown occurred, compared to r = 0.946 with lockdown. This indicates that spatial abundance of mosquitoes becomes a stronger determinant of dengue incidence when human mobility is reduced.” It doesn’t seems enough difference to indicate the latter statement.

Response: We have moderated the statement to the following (lines 197-199):

*“This indicates that spatial abundance of mosquitoes may be a slightly stronger determinant of dengue incidence when human mobility is reduced.”*

We added uncertainty intervals to the estimates to indicate the variability between simulations.

I suggest to include values related to statistical significance when spearman correlation coefficient is indicated (p-value for example). Correlation coefficient indicates strength and direction of the correlation but it is not necessarily statistically significant.

Response: While we would agree with your point for empirical studies, calculation of p-values is not usually appropriate for modeling studies in which the number of replicate simulations is at the discretion of the modeler. This is because p-values decrease as the sample size increases. Thus, when the sample size can be increased arbitrarily, this makes the p-value also arbitrary.

Line 213: it is not clear where the value of “11% of all residential locations” comes from. Seems a little lower which, in turn, can tend to underestimate the blood-meal estimates.

Response: This comes from there being 88,323 residential locations in Iquitos (not all locations are residential). We added this number to the text to clarify.

Reviewer #3: See general comments below

**Conclusions**

**Reviewer #1**: The conclusions are robust and testable by data in the future. The limitations are stated in the manuscript and the effects of COVID-19 mitigations on other infectious diseases are of interest to a wide range of researchers and of high public health relevance.

Response: Thank you very much for the positive comments.

**Reviewer #2**: Conclusions are well supported. Strengths and limitations are well described.

Response: Thank you very much for the positive comments.

I suggest a better and more complete explanation about why there is a markedly increase in infections at homes. It is understood that people spend more time at homes but still it is important the movement of people for the arrival of the virus into the house. There are reports where staying at home during the day was protective against the disease (for example Cordeiro, R., Donalisio, M.R., Andrade, V.R. et al. Spatial distribution of the risk of dengue fever in southeast Brazil, 2006-2007. BMC Public Health 11, 355 (2011). <https://doi.org/10.1186/1471-2458-11-355>). This is also important at the light of poor explanation about changes of mosquito behavior.

Response: We agree that if human movement is reduced, there will be less opportunity for transmission between households. In this case, it is mosquito movement which is driving the inter-household transmission. See for example Figure 5B – if mosquitoes do not move between households, or move at a reduced rate, then indeed, incidence does decline under lockdown. But when mosquito movement is at a moderate level like what we expect in reality, incidence is able to slightly increase. This suggests that increased intra-household transmission, coupled with a sufficient level of mosquito movement to generate inter-household transmission, combine to slightly increase incidence of infection.

We added a paragraph to the discussion to describe this (lines 314-316)

*“Mosquito movement between houses seems particularly important to enable some inter-household transmission in the context of reduced human mobility – if mosquitoes moved less, the effect of lockdown would in fact be to reduce transmission overall.”*

Reviewer #3: The conclusions will need to updated after major revisions of the study design. See general comments below

**Editorial and Data Presentation Modifications?**

**Reviewer #2:** Line 163: possibly the fig 3 is wrongly referenced.

Response: Correct, it should be Fig 2. This has been rectified.

Line 262: It is better if you explain the unit of the number 0.2

Response: This is a probability and so does not have a unit. We clarified the meaning of this in the text (line 287).

*“…; i.e. each day, each mosquito moves to a new house with probability 0.2”*

**Reviewer #3:** See general comments below

**Summary and General Comments**

**Reviewer #2**: This is a good piece of work explaining how current issues influence old issues. It is well written and the most speculative part (such as the amount of compliance of the lockdown) are well addressed.

Response: Thanks.

**Reviewer #3**: This study seeks to understand the impact of the COVI-19 pandemic on dengue transmission. This is a scenario based study looking at the the impact of lockdown via decrese human mobility have on DENV burden. This is timely and a major short/ long term concern in a pandemic context. However I have major concerns with the syudy design and thus the uselfullness of the simulations presented.

Response: Thanks. We attempted to address your specific concerns below.

Specific comments:

Why simulate 70% lockdown as ad hoc? Even though you do sensitivity analysis around this value, Are the reduction in movement realistic reductions? Why not use data driven reductions? Also we know that individual response to lockdown is heterogenous. It would be good to see actual movement data for the region used, these data while not easily available before the pandemic, are now widely available (eg., facebook mobility data, Apple, google). Reference 30 is from 2014 as such would only serve as baseline movement pre pandemic. These crude homogenous decreases end up being over -under estimations of the actuall patterns of movement.

Response: In this study, we were attempting to understand theoretically what the effect of changing mobility might be on dengue in a city environment. We were not trying to precisely, or even approximately, recreate the specific situation in Iquitos. Instead, we are using a model which happens to be based on Iquitos to explore some general effects of reduced mobility. In this sense the specific value of 70% is not important, but rather serves as a high baseline, making it easier to observe any effect. By exploring how our results change when this value changes in the sensitivity analyses, we are also able to observe any non-linearity in how dengue incidence responds to these changes in movement. In response to this comment, we added a line about changes in mobility in Iquitos to the discussion (line 342-343).

*“On the other hand, Google mobility data shows a 30% increase from baseline in household mobility, suggesting substantial changes in mobility patterns.”*

Regarding heterogeneity, there is spatial and temporal heterogeneity in who stays home, but this is not geographically structured. We added some further discussion on the topic of heterogeneity to the discussion (lines 363-366).

*“Spatio-temporal heterogeneity in compliance with lockdown may also impact dengue transmission. While we modeled some heterogeneity by randomly choosing who will comply with lockdown, there is no spatial or temporal structure to this aspect of our approach. If certain districts within a city have lower compliance with lockdown, there may be a reduced effect of lockdown in those districts.”*

In Peru About 70% of the employed population work in the informal sector, which is one of the highest rates in Latin America. These jobs are by their nature unpredictable and often in environments making social distancing difficult. Peruvians who went out to work had to use public transport, and to sell goods in very crowded markets. Even during lockdown. So your 70% baseline compliance seems to be a gross over estimation.

Response: These are good points, and may well be true. As previously mentioned, however, this study is not about trying to piece together exactly what happened in Iquitos, so much as exploring general patterns. Having a high level of compliance allows us to see these patterns more clearly, and the sensitivity analysis allows us to see the effects of other values.

We mention in the discussion that compliance was likely to have been low in Iquitos (line 341)

The latest National Household Survey suggests 11.8% of poor households in Peru live in overcrowded homes. So I wonder if your household transmission may nee dto be reasssed?

Response: We appreciate this suggestion, but we do not believe so. Our model includes realistic distributions of household sizes – see Perkins *et al.*, 2019, PLOS CB for more details – bases on aggregate demographic data for Peru and empirical data from Iquitos about household composition.

We added the following line to the methods to explain this (lines 100-101):

*“The distribution of household sizes matches the demography observed in population surveys”*

Is there evidence for the decreased vector control scenario? When we considered that lockdown measures could disrupt regular, city-wide vector control campaigns, the increase in incidence was more pronounced than with lockdown alone, especially if lockdown occurred at the optimal time for vector control.

Response: A number of researchers have raised concerns about vector control being disrupted – we refer to this in lines 64-65. Our aim was to explore the potential impact of these concerns – we are not suggesting that vector control was interrupted in Iquitos. Moreover, there have been reports of vector control being disrupted in other locations; e.g., in French overseas territories [https://journals.plos.org/plosntds/article?id=10.1371/journal.pntd.0008716#pntd.0008716.ref012](https://journals.plos.org/plosntds/article?id=10.1371/journal.pntd.0008716#pntd.0008716.ref012).

I don’t understand the point of this scenario: Initiating lockdown early in the season (July – October) what July is this? July 2020? They started lockdown in March that lasted until June. This scenario makes no sense. If it is July 2019 makes even less sense.

Response: Our point here is not to specifically model what happened this year in Iquitos, but rather to explore general principles for how lockdown might affect dengue transmission. So, it is neither July 2019 nor July 2020, but chosen to represent a lockdown late in the dengue season. We vary the month in which lockdown occurs in order to explore a range of different situations, and we choose exemplar seasons from the past in Iquitos in order to model realistic patterns of transmission.

Also heterogeneous startegies between regions in Peru outside of iquitos? Why not add a few mor regions?

Response: We explored the effect of different levels of compliance and different contact tracing strategies in the supplement – these analyses help elucidate the effect of different situations. As we were trying to understand the theoretical impact of changes in mobility and vector control, we refrained from modeling specific strategies of any locations.

Also wouldn’t stay at home scanerios be accompanied by increased portectionin the homes? Why assume nothing changes?

Response: Because we are trying to isolate the effect of reduced mobility, we did not model this. We do raise this point in the discussion as a factor mitigating the effect of reduced vector control. We also added a line (376-378) to the discussion to highlight that it would also reduce the effect of reduced mobility itself.

*“This would mitigate the impact of reduced vector control activities and reduce the impact of changes in mobility on dengue transmission.”*

If there is evidence of reduction on vector control campains (I dindt find any) Should perhaps instead of a all or none control campaign, a spectrum of coverage be explored?

Response: There is some emerging evidence that vector control has been interrupted (see the above reference from French overseas territories). We explored differing levels of vector control coverage in our previous study, and observed a fairly linear relationship between incidence of DENV infections and vector control coverage (Cavany et al., 2020, PLOS CompBiol; Fig. 10). For this reason, intermediate coverage levels would simply imply an intermediate effect of vector control, not a qualitatively different one. Moreover, it seems likely that a vector control campaign would either be canceled (due to a lockdown order) or would not be, suggesting that an all or none campaign may actually be the realistic one.
