# Supplementary material for: Pandemic-associated mobility restrictions could cause increases in dengue virus transmission
Source: PLoS Negl Trop Dis. 2021 Aug 9;15(8):e0009603. doi: 10.1371/journal.pntd.0009603 (PMC8375978; doi:10.1371/journal.pntd.0009603)
Supplement: Attachment — Submitted filename: shelter_in_place_dengue_PLOS_NTDs_response_to_reviewers_2.docx [file pntd.0009603.s013.docx]

**Editors’ comments**

Hello, in addition to the reviewers, both handling editors have reviewed the manuscript, comments, and revisions. We feel that the manuscript is meritorious, but there is still some minor comments to address. Specifically, we would suggest that it be explicitly stated in both the introduction and discussion that while the manuscript is informed by Iquitos data, this is a generalized look at hypotheticals. We further suggest that major differences in the modeled scenarios vs. what actually happened in Iquitos be addressed somewhere in the manuscript. - RCC

We thank the editor and reviewers for their comments.

- We added the following text to the introduction and discussion to emphasize that this is a modeling of hypotheticals, not of Iquitos directly (lines 88-92 and 335-338).

“Rather than seeking to model the actual patterns of dengue in Iquitos in 2020, our study uses recreations of past dengue seasons, and asks the hypothetical question of what these seasons would have looked like with changes in mobility similar to those we might expect under lockdown, or with disrupted vector control campaigns. This enables us to isolate the effect of those modifications and to present general principles from our assessments.”

“We used hypothetical scenarios with altered mobility patterns and vector control campaigns, which were not intended to directly represent the reality of what happened in Iquitos or any other city in 2020, but to gain mechanistic understanding of the effect of these type of mobility changes.”

- We compare what happened in Iquitos with our modeled situations in a paragraph in the discussion (lines 330-354).
- We highlight some differences between what happened in Iquitos and our simulations in our methods (lines 116-122):

“In reality, importation patterns almost certainly decreased during lockdown. However, we used baseline importation patterns because epidemic timing in our model is driven by the importation time series. Moreover, our aim was to isolate the effects of mobility changes and disruptions in vector control, and for these aims the baseline importation patterns served as a valid baseline. Other differences between what actually happened in Iquitos and our simulated scenarios likely include the length and level of compliance with lockdown – these differences are discussed in more detail in the Discussion.”

Specific concerns are addressed below. Line numbers refer to the tracked changes version.

Please consider modifying the title so as not to overstate the association with COVID-19, explicitly, but rather to reflect that the model assesses a hypothetical lockdown scenario (e.g., in response to the COVID-19 pandemic). - JVC

- We modified the title to **“****Pandemic-associated mobility restrictions could cause increases in dengue virus transmission”**
- We also moderated the abstract slightly so as to not overstate the COVID-19 association.

**Reviewer's Responses to Questions**

**Methods**

**Reviewer #1**: addressed my questions and methods adhere to criteria

**Reviewer #2:** The hypothesis is clear and the study design are adequated.

It is evident that the city of Iquitos is well known and that has been characterized for long time. This can be seen through the developed model on this city. It is necessary to read other papers to fully understand the model, but the focus of the paper is not the development of the model, so it is not a big problem.

**Reviewer #3**: See general comments

**Results**

**Reviewer #1**: yes to all criteria

**Reviewer #2**: Results do match the analysis plan.

Results are now more clearly presented.

**Reviewer #3**: See general comments

**Conclusions**</b></br></br>

**Reviewer #1**: yes to all criteria

**Reviewer #2**: Conclusions are well supported. Strengths and limitations are well described.

Just a little concern: Most of simulations were done on the “serotype invasion” season, which were the one where lockdown had an effect on incidence. There is no evidence that a serotype invaded Iquitos during the 2020 (or it is not stated in the manuscript).

At the end of the manuscript, the idea that lockdown has an effect on dengue incidence remains. So, conclusions must specify that the effects seen on most of simulations along this study apply in that especific season. For the other two scenarios, probably the most likely to have occurred, no effect was seen due to lockdown, according to the first simulations (and Fig 1). I know that in each experiment it is stated the season where experiments were runned, but I would like to suggest that be sure that correct idea remains at the end of the document.

- We clarified in both the abstract and the discussion that effects were seen in seasons in which a new serotype invaded (lines 22 and 304-305).

**Reviewer #3**: See general comments

**Editorial and Data Presentation Modifications?**

Use this section for editorial suggestions as well as relatively minor modifications of existing data that would enhance clarity. If the only modifications needed are minor and/or editorial, you may wish to recommend “Minor Revision” or “Accept”.

Reviewer #1: (No Response)

Reviewer #2: None

Reviewer #3: See general comments

**Summary and General Comments**

**Reviewer #1**: The authors did a good job of addressing my questions and comments.

**Reviewer #2:** This is a good piece of work and is well written. It is appropriate for the current times and the situation. It is a well use of a previously developed model.

**Reviewer #3:** The authors study the impact of COVID-19 measure on dengue transmission using an ABM with mobility and vector control.

While the simulation study is potentially interesting, I still have major issues with the assumptions in the model particularly the choices for lockdown stringency and duration.

Below I make more specific comments on my issues.

Below we grouped reviewer 3’s comments in ways that we hope will help address similar topics.

Assumptions of the model

I still have major issues with the methodology under the lockdown.

The authors mention that “Transmission in the model is partially driven by time series of imported infections, which were calibrated to estimates of the time-varying, serotype-specific force of infection over an 11-year time period.” Is this still valid in the lockdown?

- We agree with the reviewer that importation patterns are likely to have changed under lockdown. However, in this paper, we wanted to isolate the effect of mobility changes and disruption to vector control on DENV transmission. On lines 399-404 we explain that we did not change importation patterns as a limitation of our approach.
- One of the reasons we chose not to look at importation patterns is that our model is fairly heavily driven by importation patterns, in particular to match the timing of past epidemics. This is a known limitation of the model. We added the following text to the methods (lines 116 – 122) to clarify this point:

“In reality, importation patterns almost certainly decreased during lockdown. However, we used baseline importation patterns because epidemic timing in our model is driven by the importation time series. Moreover, our aim was to isolate the effects of mobility changes and disruptions in vector control, and for these aims the baseline importation patterns served as a valid baseline.”

“See Fig 3 in Perkins et al. and S1 Fig in Cavany et al. for a visual representation of the calibration” Why not just have the figure in SI? This seems a little lazy…

- We added the figure showing the calibration to the SI (S1 Fig) and added text to the methods (line 114) directing the reader to this figure.

“Movements… those that might be expected under lockdown” Was this validated with any type of mobility data?

I had this same comment the first round of submission: How did you arrive at the 70% staying home? This is awfully high? In Peru About 70% of the employed population work in the informal sector, which is one of the highest rates in Latin America. These jobs are by their nature unpredictable and often in environments making social distancing difficult. Peruvians who went out to work had to use public transport, and to sell goods in very crowded markets. Even during lockdown. So your 70% baseline compliance seems to be a gross overestimation.

There were some 2-week period lockdowns enforced with the last one in January 2021, but no record of such a stringent number.

Line 257: “In all baseline analyses, we used values of 70% compliance with lockdown orders and a lockdown length of three months.” I still don’t understand why this is the baseline. This is not realistic.

While I appreciate the sensitivity analysis that I had requested, I still think that the baseline should be the actual length and the actual dates of lockdowns. Also why not use information of dengue in this season?

Why is the lockdown 3 months as the baseline? This is not what happened?

- Rather than seek to recreate what happened in Iquitos, we are using a hypothetical approach to understand some general principles of how these changes affect DENV transmission. We added text to the introduction and discussion to better explain the hypothetical nature of our approach (lines 88-92 and 335-338).

“Rather than seeking to model the actual patterns of dengue in Iquitos in 2020, our study uses recreations of past dengue seasons, and asks the hypothetical question of what these seasons would have looked like with changes in mobility similar to those we might expect under lockdown, or with disrupted vector control campaigns. This enables us to isolate the effect of those modifications and to present general principles from our assessments.”

“We used hypothetical scenarios with altered mobility patterns and vector control campaigns, which were not intended to directly represent the reality of what happened in Iquitos or any other city in 2020, but to gain mechanistic understanding of the effect of these type of mobility changes.”

- It would be a much larger, and totally different, project if we were to attempt to model what happened in 2020-2021. To undertake that kind of study, we would have to model the period 2010-2020 in order to get the correct immunity background. To do that, we would then need data from serological surveys and mosquito household surveys from that time period. Then we would need to use the serological surveys to re-estimate the incidence of infection in that time period and the household surveys to re-estimate the spatiotemporal patterns of mosquito abundance. We would then need to re-calibrate the full model to the re-estimated incidence of infection. Even if we did all that, these data are currently not yet available for the time period in question for Iquitos. Thus, from a practical perspective we would not be able to do such a study at this time. In years to come, once the necessary data become available, such a study could be done and prove valuable. While we don’t dispute the value such a study would have, it was not the one we did, and we feel that it would be most appropriate for the reviewer to assess the study we did rather than propose a different study. Instead, we made our best effort to use the tools available to us to address a pressing question of critical importance. We aimed to do so in a timely manner at a time when there was a pressing desire for such answers. Our use of hypothetical seasons allows us to gain insight into this question while leaving open the question of precisely what impact these changes had in practice in Iquitos, or perhaps by extension in any other city.
- We added text to the discussion to describe what would be required to assess what actually happened in Iquitos, and to highlight the potential value such a future study would have.
- We added some text to justify the choice of 70% as a baseline:

“We chose 70% as a baseline to reflect large changes in mobility observed in Peru and other countries around the time of the first lockdowns in 2020. We chose a relatively high value to help use better assess the relative effect of the changes. We explored other values of compliance in a sensitivity analysis”.

I don’t think this paper shows useful simulations…. The choice of seasons is weird, “this back in time” is slightly pointless as these seasons were affected by mobility, weather and other factors of those years.

Because I don’t understand the baseline and the choice of season, I have trouble believing the results.

- We respect that the reviewer does not find our approach convincing. We feel, however, that our use of past seasons as hypotheticals is a valid approach for addressing the goals of the study, and contributes important new insights. We chose to use past seasons because we know that these are reflective of plausible situations given the environmental conditions, importation patterns, mobility patterns, and other relevant factors in Iquitos. Taking this approach, we are able to control for other factors and isolate the effect of mobility changes.
- While we agree that mobility patterns did affect outcomes during past seasons, it seems unlikely that they would change substantially from year-to-year prior to 2020. They, therefore, are suitable baseline to understand the effect of changes in mobility.
- Understanding the effect of changes in mobility patterns on DENV transmission is a difficult problem. Some papers, referred to in the manuscript, assessed whether actual changes in dengue incidence in 2020 could be associated with lockdown measures. While such papers do a good job of controlling for changes in other drivers of DENV transmission, the complexity of DENV transmission makes this an inherently exceedingly difficult problem. Additionally, those papers are purely statistical in nature, and describe associational findings. Our approach, which controls for other drivers, provides a complement to prior approaches, and is to our knowledge the first study to explore possible mechanisms of these changes using a mechanistic model. From our perspective, as long as they are clearly explained, both approaches are valid, and both help to further our understanding of how lockdown mobility changes affect DENV transmission.
- To further emphasize the hypothetical nature of our approach, we added text to the introduction and discussion explaining that our results are indeed hypothetical in nature and are not intended to describe precisely the epidemic in Iquitos in 2020 (lines 88-92 and 335-338).

Line 219: “According to the most recent available information, there are 92,896 buildings in Iquitos.” Why not cite where this information came from?

- This information came from reports of past city-wide spraying campaigns which recorded the number of households in each zone of the city, and the number sprayed. These data are not publicly available, and were obtained through personal communication with our collaborators Amy Morrison and Helvio Astete. We added text to the manuscript to explain this and refer to a paper where this approach is explained in more detail (line 232-234).

I’m also confused as to why under lockdown the number of people per house is more homogeneous. Shouldn’t we except this to actually be the opposite? Since these regions vary widely in terms of socio-demography. The latest National Household Survey suggests 11.8% of poor households in Peru live in overcrowded homes. So I wonder if your household transmission may need to be reassessed?

- Household sizes in our model reflect actual demography in Peru, including overcrowding. We are confident that we are accurately capturing this aspect of demography in the city.
- The number of people under lockdown is more homogeneous compared to a baseline in which people move around more, because there are many fewer visitors to homes and people spending time in homes are primarily just its residents. Hence, it makes sense that heterogeneity would decrease as fewer people would be likely to congregate in one place under lockdown.

Im pasting comments from my first round review that I feel still haven’t been address:

Why not use data driven reductions? Also we know that individual response to lockdown is heterogeneous. It would be good to see actual movement data for the region used, these data, while not easily available before the pandemic, are now widely available (eg., facebook mobility data, Apple, google). Reference 30 is from 2014 as such would only serve as baseline movement pre pandemic. These crude homogenous decreases end up being over -under estimations of the actual patterns of movement.

- We added text to the discussion to compare our changes to changes in the most relevant Google metrics. These metrics indicate substantial changes in mobility during the first lockdown of 2020, in both Peru in general and in Loreto specifically. Unfortunately, using these data to directly inform lockdown compliance is complicated because they do not reflect the same things.
- In terms of heterogeneity, these data would not inform estimates of heterogeneity in our model, because our model is of a city. The resolution of these metrics is more coarse than a city. It covers a much broader and geographically diverse area.
- We would like to reiterate that we are not attempting to model precisely what happened in Iquitos. Instead we aimed to examine effects on general patterns of virus transmission, under hypothetical scenarios.

Further, there is high heterogeny is compliance between regions and so this blanket compliance seem unrealistic. The authors argue that a major strength is the study is the spatial component, yet I don’t see any sensible calibration in this regard. On the contrary.

- For reasons explained above it would be very difficult for us to calibrate spatial heterogeneity in compliance.
- We added text to better explain our inability to model structured heterogeneity in changes in mobility as a limitation on lines 411-414.

“Fifth, in order to simplify the analysis we did not incorporate structured spatial heterogeneity in compliance. While we do model heterogeneity in compliance between individuals, structured differences between regions may lead to effects not captured in our analysis.”

Last time I also asked If there is evidence of reduction on vector control campaigns (I didn't find any) Should perhaps instead of all or none control campaigns, a spectrum of coverage be explored?

- As previously stated, there is at least one report that vector control campaigns were disrupted (in French overseas territories, see e.g. [https://journals.plos.org/plosntds/article?id=10.1371/journal.pntd.0008716#pntd.0008716.ref012](https://journals.plos.org/plosntds/article?id=10.1371/journal.pntd.0008716#pntd.0008716.ref012)). Moreover, the potential for disruption was a significant concern of mosquito control and public health specialists, and was raised in several commentary papers, some of which we cite in the introduction to our manuscript. In our view, this concern makes modeling this situation a valid and interesting question.
- We explored differing levels of vector control coverage in our previous study and observed a fairly linear relationship between incidence of DENV infections and vector control coverage (Cavany et al., 2020, PLOS CompBiol; Fig. 10). For this reason, intermediate coverage levels would simply imply an intermediate effect of vector control, not a qualitatively different effect.
- It seems likely to us that a vector control campaign would either be canceled (due to a lockdown order) or would not be canceled, consistent with the idea that an all or none campaign is realistic. In this regard, we disagree with the reviewer’s assertion that a range of coverage is necessarily more realistic.
